# Comprehensive Identification of Fim-Mediated Inversions in Uropathogenic Escherichia coli with Structural Variation Detection Using Relative Entropy

**DOI:** 10.1128/mSphere.00693-18

**Published:** 2019-04-10

**Authors:** Colin W. Russell, Rashmi Sukumaran, Lu Ting Liow, Balamurugan Periaswamy, Shazmina Rafee, Yuemin C. Chee, Swaine L. Chen

**Affiliations:** a Department of Medicine, Yong Loo Lin School of Medicine, National University of Singapore, Singapore; b Infectious Disease Group, Genome Institute of Singapore, Singapore; c GERMS platform, Genome Institute of Singapore, Singapore; University of Kentucky

**Keywords:** type 1 pili, genomics, information theory, phase variation, structural variations, uropathogenic *Escherichia coli*

## Abstract

Most urinary tract infections (UTIs) are caused by uropathogenic Escherichia coli (UPEC), which depends on an extracellular organelle (type 1 pili) for adherence to bladder cells during infection. Type 1 pilus expression is partially regulated by inversion of a piece of DNA referred to as *fimS*, which contains the promoter for the *fim* operon encoding type 1 pili. *fimS* inversion is regulated by up to five recombinases collectively known as Fim recombinases. These Fim recombinases are currently known to regulate two other switches: the *ipuS* and *hyxS* switches. A long-standing question has been whether the Fim recombinases regulate the inversion of other switches, perhaps to coordinate expression for adhesion or virulence. We answered this question using whole-genome sequencing with a newly developed algorithm (structural variation detection using relative entropy [SVRE]) for calling structural variations using paired-end short-read sequencing. SVRE identified all of the previously known switches, refining the specificity of which recombinases act at which switches. Strikingly, we found no new inversions that were mediated by the Fim recombinases. We conclude that the Fim recombinases are each highly specific for a small number of switches. We hypothesize that the unlinked Fim recombinases have been recruited to regulate *fimS*, and *fimS* only, as a secondary locus; this further implies that regulation of type 1 pilus expression (and its role in gastrointestinal and/or genitourinary colonization) is important enough, on its own, to influence the evolution and maintenance of multiple additional genes within the accessory genome of E. coli.

**IMPORTANCE** UTI is a common ailment that affects more than half of all women during their lifetime. The leading cause of UTIs is UPEC, which relies on type 1 pili to colonize and persist within the bladder during infection. The regulation of type 1 pili is remarkable for an epigenetic mechanism in which a section of DNA containing a promoter is inverted. The inversion mechanism relies on what are thought to be dedicated recombinase genes; however, the full repertoire for these recombinases is not known. We show here that there are no additional targets beyond those already identified for the recombinases in the entire genome of two UPEC strains, arguing that type 1 pilus expression itself is the driving evolutionary force for the presence of these recombinase genes. This further suggests that targeting the type 1 pilus is a rational alternative nonantibiotic strategy for the treatment of UTI.

## Abstract (precis)

UTI is a common ailment that affects more than half of all women during their lifetime. The leading cause of UTIs is UPEC, which relies on type 1 pili to colonize and persist within the bladder during infection. The regulation of type 1 pili is remarkable for an epigenetic mechanism in which a section of DNA containing a promoter is inverted. The inversion mechanism relies on what are thought to be dedicated recombinase genes; however, the full repertoire for these recombinases is not known. We show here that there are no additional targets beyond those already identified for the recombinases in the entire genome of two UPEC strains, arguing that type 1 pilus expression itself is the driving evolutionary force for the presence of these recombinase genes. This further suggests that targeting the type 1 pilus is a rational alternative nonantibiotic strategy for the treatment of UTI.

## INTRODUCTION

Uropathogenic Escherichia coli (UPEC) is the primary cause of urinary tract infections (UTIs) (1, 2), which are estimated to affect more than half of all women during their lifetime (3). The total annual cost of community-acquired and nosocomial UTIs in the United States was estimated to be $2 billion in 1995 (3). Although UTIs have traditionally been effectively treated with antibiotics, in some patients UTIs recur despite apparently appropriate antibiotic therapy and sterilization of the urine (4). Furthermore, UTIs are the first or second most common indication for antibiotic therapy (5, 6), making them a major contributor to rising antibiotic resistance rates (7). Therefore, substantial effort has been devoted to studying the molecular mechanisms by which UPEC cause UTI in the service of developing alternative preventive and therapeutic strategies (2, 8–11).

One of the major successes in UTI research has been the recognition of the importance of type 1 pili for causing UTI (12–14). Type 1 pili, encoded by the *fim* operon, are hair-like, multiprotein structures that extend from the outer membrane and terminate in the adhesin protein FimH (15–17). FimH binds to mannose residues on glycosylated bladder surface proteins such as uroplakin protein UPIa (18) and α3β1 integrin heterodimers (19). Adhesion to the bladder epithelium can lead to internalization of the bacteria into host cells and formation of intracellular bacterial communities (IBCs) (20–23). Bacteria in IBCs are protected from the immune response and antibiotic treatment and can later escape from the host cells to cause recurrent infection (24, 25). Therefore, type 1 pili directly contribute both to the initiation of infection and to intracellular persistence. Several new strategies have focused on blocking the function of type 1 pili by small-molecule inhibition or vaccination (26, 27).

The pilus structural proteins (including the FimH adhesin) and the chaperone-usher proteins that mediate pilus biogenesis are encoded within the *fimAICDFGH* operon (15, 16). Regulation of type 1 pili expression centers on the epigenetic alteration of the *fim* operon promoter, which is located within the invertible *fim* switch *fimS* (28, 29). When *fimS* is in the ON orientation, the promoter is positioned to transcribe the *fim* genes and type 1 pili may be synthesized. In contrast, when the *fimS* promoter is in the OFF orientation, bacteria do not produce type 1 pili.

Switching of *fimS* from one state to another is regulated by recombinases which bind to inverted repeat (IR) sequences that flank the switch. Two recombinases, FimB and FimE, are encoded by genes that are genetically linked to the *fim* operon and *fimS* switch (30). Other known recombinases acting at *fimS* include the genetically unlinked IpuA and FimX (30–32). Interestingly, both the linked and unlinked Fim recombinases are also able to mediate the inversion of other switches. The *hyxS* switch is inverted by FimX (33), while *ipuS* was shown to be inverted by FimE, FimX, IpuA, and IpuB (but not FimB) (34). Like for *fimS*, inversions of *hyxS* and *ipuS* appear to regulate downstream gene expression, but the full importance of these genes in pathogenesis is still not clear.

An open question in the field has been whether the Fim recombinases are utilized in the regulation of other, still unknown switches and whether such switches may be related to pathogenesis. To search for novel invertible elements, we developed an algorithm named structural variation detection using relative entropy (SVRE) to detect genomic structural variations (SVs) in whole-genome sequencing data. We applied SVRE to uropathogenic strains overexpressing each Fim recombinase. In addition to the known inversions at *fimS*, *hyxS*, and *ipuS*, SVRE detected several SVs that were recombinase independent. Importantly, no new invertible switches were found, indicating that *fimS* is inverted by several recombinases that regulate little else, suggesting that tuning of type 1 pilus expression is of strong evolutionary importance.

## RESULTS

### Development of SVRE.

Invertible sequences like *fimS* are one class of SV, which also includes deletions, duplications, translocations, and more complex rearrangements. Several programs have been developed to call SVs from whole-genome sequencing data. One primary strategy for SV detection is to identify paired-end reads with unusual mapping patterns. Generation of DNA libraries for next-generation sequencing typically includes a size selection step that restricts the physical size of the DNA fragments that are carried forward for sequencing. When mapped to an ideal reference genome, the distance between paired-end reads should reflect this length. Additionally, the reads should map to opposite strands of the genome. Paired-end reads with an appropriate mapping distance and read orientation are termed “concordant” reads. In contrast, in the presence of an SV in the input DNA relative to the reference genome, paired-end reads associated with the SV map at a distance or orientation that differs from this expectation; these reads are called “discordant” reads.

**FIG 1:**
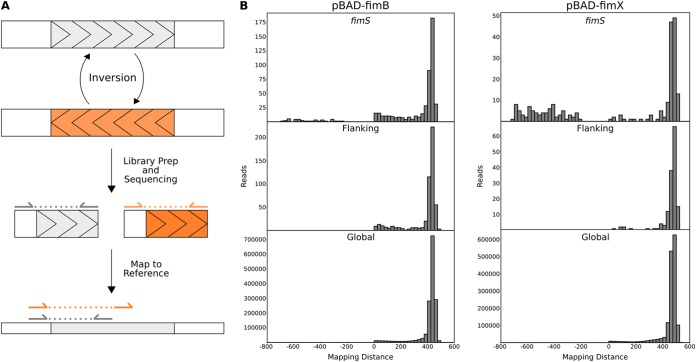
Detection of the *fimS* inversion by the SVRE algorithm. (A) A schematic of how inversions are detected by SVRE. Under the right experimental conditions, invertible elements are present in both orientations (shaded gray and orange). After library preparation and sequencing, paired reads derived from sequence in the reference orientation will map to opposite strands of the reference genome with the expected mapping distance. In contrast, paired reads derived from inverted sequences will map to the same strand of the reference genome, resulting in a negative mapping distance, which may also be of an unexpected magnitude. (B) UTI89 carrying a plasmid encoding an arabinose-inducible *fimB* or *fimX* gene was sequenced and analyzed using SVRE. Mapping distance distributions are displayed for windows associated with *fimS* and determined by SVRE to have a significant distribution deviation, windows flanking *fimS*, and the global distribution.

We developed SVRE, an algorithm that detects SVs by analyzing the distribution of mapping distances in segments of the genome. When reads span an SV, the local mapping distances for these reads should follow a different distribution based on the type of SV; the difference in distribution is generated by discordant reads. In the case of an invertible element like *fimS*, the genomic material used for sequencing may contain a mixture of both orientations (Fig. 1A). Reads derived from the invertible element map to the reference genome differently depending on the orientation of the element. If the orientation is the same as the reference, the reads will align with the expected mapping distance to opposite strands (gray arrows in Fig. 1A). However, if the orientation is reversed, the paired-end reads will map to the same strand and with a mapping distance different from that selected during library preparation (orange arrows in Fig. 1A). When paired-end reads map to the same strand, SVRE assigns them a negative mapping distance. Therefore, a hallmark of inversions is a local mapping distribution that skews toward negative values.

SVRE compares the local mapping distribution of each genome segment to the global distribution, which includes the mapping distances of all paired-end reads genome-wide. The comparison of local and global mapping distributions is made using relative entropy, a statistical test derived from information theory (35). By using relative entropy, SVRE improves on existing SV detection software by providing a more general theoretical foundation for detecting anomalous insert length distributions (as opposed to assuming a normal distribution), resulting in improved signal-to-noise ratio and accuracy. Full theoretical and algorithmic details for SVRE can be found in Materials and Methods and Text S1 in the supplemental material.

10.1128/mSphere.00693-18.1 TEXT S1 Description of how the SVRE program is implemented, including how relative entropy is calculated. Download Text S1, PDF file, 0.09 MB. Copyright © 2019 Russell et al. 2019 Russell et al. This content is distributed under the terms of the Creative Commons Attribution 4.0 International license.

### Application of SVRE to discover SVs in UTI89.

SVRE was applied to the uropathogenic strain UTI89 carrying a pBAD33-based plasmid providing arabinose-inducible overexpression of *fimB* or *fimX*, both of which bias the *fimS* switch toward the ON orientation (a strategy similar to that used in the work described in reference 33). In contrast, the UTI89 reference genome has the *fimS* switch in the OFF orientation; therefore, induction of *fimB* or *fimX* should result in a structural variation (inversion) at *fimS* relative to the published reference sequence. Indeed, with overexpression of either recombinase, windows associated with the *fim* switch showed a local mapping distance distribution that differed from the global distribution (Fig. 1B). The difference in the distributions can be attributed primarily to the negative mapping distances observed around the *fim* switch due to paired reads mapping to the same strand, indicative of an inversion. The distribution in flanking windows not associated with *fimS* was similar to the global distribution, and these windows were not predicted by SVRE to contain an SV (Fig. 1B).

**FIG 2:**
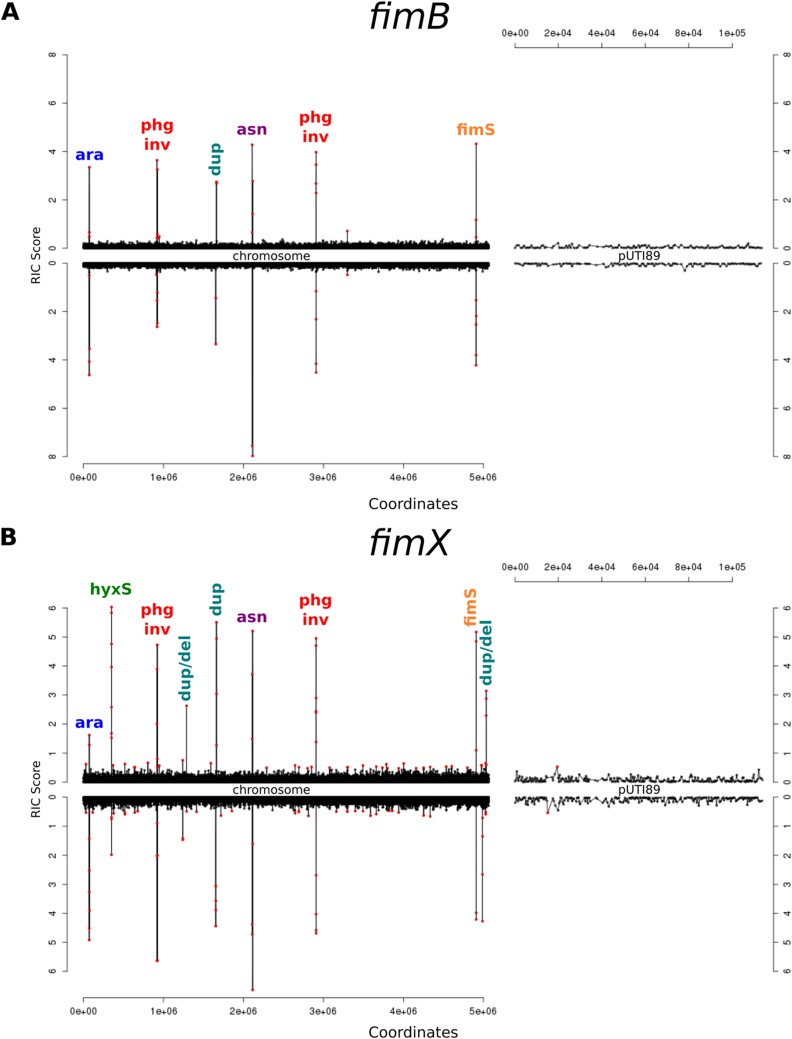
Detection of known and novel structural variations by SVRE in UTI89 overexpressing recombinases. UTI89 cells carrying a plasmid encoding an arabinose-inducible *fimB* (A) or *fimX* (B) gene were sequenced and analyzed using SVRE as in Fig. 1. Relative information criterion (RIC) scores are graphed for all windows on the UTI89 chromosome and the pUTI89 plasmid. Peaks are labeled according to the SV they represent as described in the text.

The SVRE algorithm assigns a relative information criterion (RIC) score (i.e., relative entropy) to each window. The RIC score peaks for the *fimS*-associated windows were distinct and well above the genomic background (Fig. 2). In addition to the *fimS* peak, there was a distinct peak at *hyxS* in the FimX sample but not the FimB sample. The detection of the *fimS* and *hyxS* peaks with recombinase overexpression demonstrated the ability of SVRE to find known SVs.

In addition to the *fim* and *hyx* switches, other genomic locations exhibited distinct peaks in RIC scores. Both samples shared a RIC score peak that corresponded to the *ara* locus (labeled “ara” in Fig. 2), which is an artifact originating from the use of pBAD plasmids. The remaining peaks included two cases of inversions occurring within prophage (labeled “phg inv” in Fig. 2), as well as one inversion occurring in an area containing three asparagine tRNA genes (labeled “asn” in Fig. 2). These inversions were predicted to occur in both the FimB and FimX samples. Both samples also shared a prediction of prophage duplication (labeled “dup”), with 2 additional cases of duplication and deletion of prophage (labeled “dup/del”) found only in the FimX sample. Using PCR, each of these SVs was validated in the *fimB*- and *fimX*-overexpressing strains, but they were also found to occur in control cells not overexpressing any recombinases (Fig. S1), indicating that these SVs do not appear to be regulated by Fim recombinases. In addition, one of the prophage-associated inversions occurred in the vicinity of a predicted prophage-encoded invertase that is homologous to other phage systems that have been shown to regulate linked prophage promoters (36). The lack of novel invertible elements regulated by FimB and FimX confirms that these recombinases are specific to *fimS* (FimB and FimX) and *hyxS* (FimX).

10.1128/mSphere.00693-18.2 FIG S1 Confirmation of novel structural variations in UTI89. PCR was used to validate SVs predicted by SVRE, using different primer configurations depending on the specific SV type. (A) For inversions, two PCRs were performed. One reaction used a set of primers that produced a band when the invertible element was in the orientation found on the reference genome. In contrast, the other reaction used a set of primers that produced a band if there was an inversion event. One primer is shared between the two primer sets (typically the primer that anneals to the invertible sequence), resulting in a total of three primers, which are depicted in panel A as arrows. A successful reaction is noted by a dashed line between primers. (B) Deletions were detected by using distant primer sets that produce a band only if the intervening sequence is deleted, bringing the priming sites closer together. (C) Duplications were detected using outward-facing primer pairs that produce a band only if a tandem duplication event occurs. While only two primers are used in this reaction, the number of possible annealing sites increases from two to four upon duplication, with two of those sites able to produce a PCR product. (D to I) For each SV detected by SVRE, the leftmost coordinates of significant windows called by SVRE are represented by red (UTI89/pBAD-fimB) and blue (UTI89/pBAD-fimE) lines on a segment of the genome. The primers used to confirm the predicted SVs are depicted as arrows on the schematic of the neighboring genes, and the gels that resulted from the use of those primers are shown below. Primers are either numbered or color-coordinated to demonstrate which primers were used in each gel. (D to F) Confirmation of inversions at 0.9 Mb (D), 2.1 Mb (E), and 2.9 Mb (F) were performed in UTI89 (Ctrl), UTI89/pBAD33 (EV), and UTI89/pBAD-fimX (*fimX*) cells. The linked phage invertase *pin* is highlighted in panel D. (G to I) Confirmation of a prophage deletion at 1.6 Mb (G) and prophage duplication and deletions at 1.2 Mb (H) and 5.0 Mb (I). The PCR was performed using WT UTI89 as well as UTI89 Δ*fimB* Δ*fimE* Δ*fimX* (ΔBEX). Download FIG S1, PDF file, 1.8 MB. Copyright © 2019 Russell et al. 2019 Russell et al. This content is distributed under the terms of the Creative Commons Attribution 4.0 International license.

### Discovery and validation of structural variations in CFT073.

The pyelonephritis isolate CFT073 contains two recombinases (IpuA and IpuB) and one known invertible switch (*ipuS*) that are not found in UTI89 (31). Although IpuB was not able to regulate *fimS*, IpuA was shown to be capable of regulating the *fim* switch both *in vitro* and *in vivo*, adding another layer to type 1 pilus regulation (31). The *ipuS* switch is located between *ipuA* and *ipuR* and was shown to be inverted by IpuA, IpuB, FimX, and FimE but not FimB (34).

**FIG 3:**
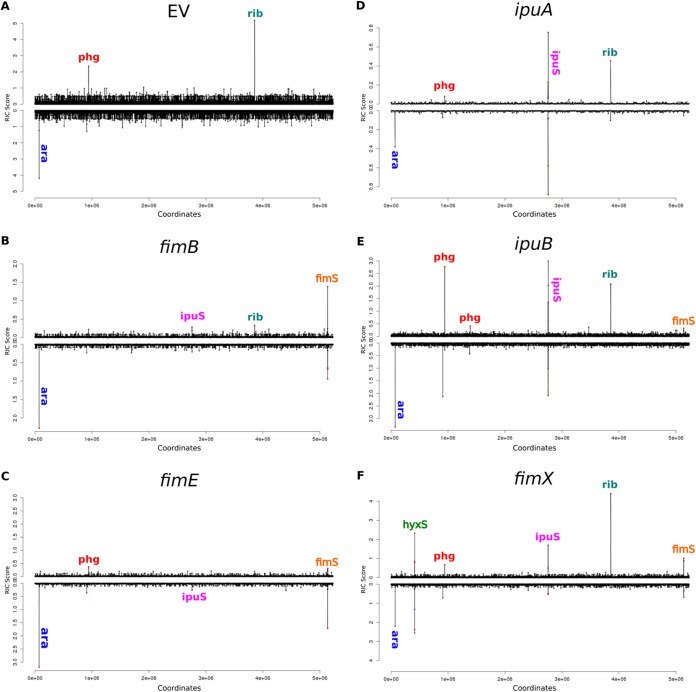
Detection of structural variations using SVRE in CFT073 overexpressing recombinases. RIC scores for all windows on the CFT073 chromosome for cells carrying the pBAD33 control plasmid (A) or cells overexpressing *fimB* (B), *fimE* (C), *ipuA* (D), *ipuB* (E), and *fimX* (F). Significant peaks are labeled according to the SV they represent as described in the text.

The CFT073 allele for each of these recombinases (in cases where they differed from UTI89) was cloned into pBAD33. CFT073 cells carrying each of these plasmids were sequenced and analyzed with SVRE (Fig. 3). As expected, a peak for *hyxS* was detected for CFT073/pBAD-fimX cells (Fig. 3F) but not for any of the other samples. Distinct peaks for *fimS* were observed for the FimB, FimE, IpuB, and FimX samples (Fig. 3B, C, E, and F). There were distinct *ipuS* peaks with overexpression of any of the recombinases (Fig. 3B to F). Similar to the case for the UTI89 samples, other peaks were observed that were unrelated to Fim recombinase activity, some of which were present in the empty-vector sample (Fig. 3A). These included the *ara* operon artifact (“ara” in Fig. 3), a false-positive peak associated with mismapping to ambiguous bases in *rrnD* (“rib”), and phage deletions and duplications (“phg”). The phage SVs were found to occur regardless of Fim recombinase expression (Fig. S2). Again, as in UTI89, there was no detection of novel invertible elements regulated by the Fim recombinases.

10.1128/mSphere.00693-18.3 FIG S2 Confirmation of novel structural variations in CFT073. Validation of SVs predicted by SVRE was completed using PCR as for Fig. S1. For each SV, the leftmost coordinates of significant windows called by SVRE are represented by red (pBAD-fimB), black (pBAD-fimE), orange (pBAD-ipuA), green (pBAD-ipuB), and blue (pBAD-fimX) lines. The primers used to confirm the predicted SVs are depicted on the schematic of the neighboring genes, and the gels that resulted from the use of those primers are shown below. Confirmation of the SVs was performed in CFT073 carrying either pBAD33 (EV) or plasmids encoding the various recombinases. (A and B) Detection of duplication and deletion of phage at 0.9 Mb (A) and a phage at 1.3 Mb (B). Download FIG S2, PDF file, 1.9 MB. Copyright © 2019 Russell et al. 2019 Russell et al. This content is distributed under the terms of the Creative Commons Attribution 4.0 International license.

### Effects of recombinase overexpression on *ipuS* inversion and expression of neighboring genes.

**FIG 4:**
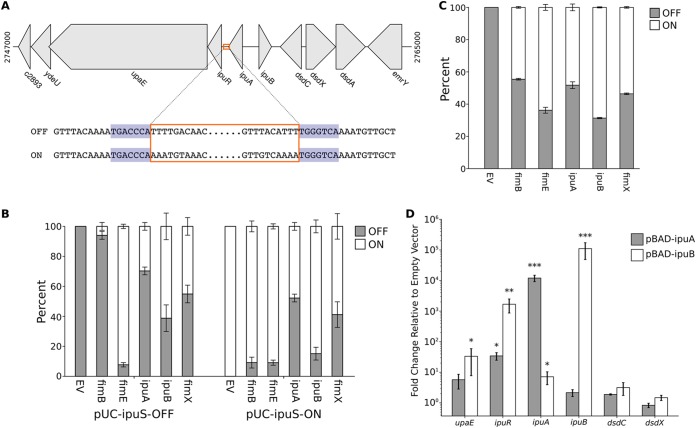
The *ipuS* switch can be inverted by any of the Fim recombinases to drive expression of *ipuR* and *upaE*. (A) A schematic of the genomic location of the *ipuS* invertible element, with *ipuS* outlined in orange and the 7-bp IRs shaded in blue. The breakpoints were determined by cloning the invertible element and surrounding sequence from CFT073/pBAD-ipuA induced with arabinose, followed by Sanger sequencing. (B) Quantification of *ipuS* orientation in MDS42 carrying pSLC-372, which contains *ipuS* in the OFF orientation, or pSLC-373, which contains *ipuS* in the ON orientation. The cells also carry a plasmid encoding one of the recombinases or an empty vector control (EV). Orientation was quantified via PCR to amplify across the switch, followed by PacI digestion, and measurement of band density using ImageJ. (C) The orientation of the *ipuS* switch was quantified as in panel B in wild-type (WT) CFT073 with induced expression of different recombinases. (D) CFT073 carrying pBAD33, pBAD-ipuA, or pBAD-ipuB was induced with arabinose and RT-qPCR was performed to quantify relative gene expression. Gene expression was normalized to 16S levels, and the expression levels are expressed relative to the pBAD33 control samples. The Δ*C_T_* values of each condition were compared to that of the pBAD33 sample using an unpaired, two-tailed *t* test. *, P < 0.05; **, *P* < 0.01; ***, *P* < 0.001. For panels B to D, bars indicate the means, with error bars representing the SEMs.

We observed an *ipuS* peak in the pBAD-fimB sample (Fig. 3B) despite previous data suggesting that FimB is not able to invert *ipuS* (34). To investigate this further, *ipuS* in the ON and OFF orientations was cloned onto a pUC19 backbone. The plasmid sequences confirmed the 7-nucleotide IRs that were observed previously (Fig. 4A) (34). Each recombinase was expressed in the MDS42 strain background (chosen due to its lack of endogenous recombinases) in the presence of the *ipuS*-OFF or *ipuS*-ON plasmids (Fig. 4B). FimB was capable of inverting *ipuS*, but it had the lowest efficiency of all the recombinases (Fig. 4B). The ability of FimB to invert *ipuS* was confirmed in CFT073 (Fig. 4C). Overall, IpuB and FimE exhibited the greatest efficiency in OFF-to-ON inversion, whereas IpuA was most efficient at ON-to-OFF inversion (Fig. 4B and C). These data demonstrate that all of the recombinases, including FimB, are capable of facilitating the inversion of *ipuS*, further validating the accuracy of the SVRE predictions.

It was previously demonstrated that the orientation of the *ipuS* switch can regulate expression of *ipuR* and *upaE* (34). It has also been hypothesized that IpuA may regulate expression of the d-serine utilization locus (37). To delineate the genes that are affected by *ipuS* inversion, reverse transcription-quantitative PCR (RT-qPCR) was used to quantify relative expression of several genes in CFT073 cells overexpressing IpuA or IpuB (Fig. 4D). No significant change of expression was observed for *dsdC* or *dsdX*, indicating that neither IpuA, IpuB, nor the orientation of *ipuS* affects expression of the d-serine utilization locus. In contrast, expression of *ipuR* was increased ∼1,600-fold with IpuB overexpression and ∼34-fold with IpuA overexpression (Fig. 4D); this correlates with the orientation of the *ipuS* promoter switch. The significant increase in *upaE* expression was not as dramatic, ∼33-fold, with IpuB overexpression. Together, these data suggest that *ipuS* inversion affects the expression only of *ipuR* and *upaE* and clarifies that *dsdC* and *dsdX* transcription is not controlled by *ipuS*.

## DISCUSSION

The *fimS* switch is a well-studied example of epigenetic regulation by DNA inversion (29, 38, 39). A single bacterium can give rise to two populations which differ only in the orientation of the *fimS* switch, and individual bacteria can convert between these two populations. The inversion of this switch was first noted to be controlled by two linked recombinases, FimB and FimE (30); in general, *fimS* inversion is described as stochastic, though regulation of the recombinases and several other proteins which bind to regions in the *fimS* switch can influence the bias (15, 38). Therefore, type 1 pilus expression exhibits phase variation (stochastic inversion) that is responsive to environmental conditions (regulation of bias). With the sequencing of the genomes of several UPEC strains, most notably CFT073 (40) and UTI89 (41), genes encoding additional recombinases with homology to FimB and FimE were discovered (31, 32). These recombinases, like FimB and FimE, were found to regulate inversion of promoter elements genetically linked to the respective recombinase gene. Interestingly, these recombinases also have activity at *fimS*, providing potentially additional layers of regulation for type 1 pilus expression (31, 32). Importantly, the inverted repeats for these known switches do not always share obvious sequence similarity (see below), implying that a simple search for similar inverted sequences in the genome is not a viable strategy for discovering other invertible switches. The discovery of these unlinked recombinases, therefore, raises several salient questions: (i) do the *fim*-linked FimB and FimE recombinases also have other inversion targets in the genome; (ii) what is the full suite of targets for all of the Fim recombinases; (iii) what is the consequence of coordinating inversion of multiple promoters with the same recombinases; (iv) are the other non-*fim* promoters important for type 1 pilus expression or function; (v) what additional control of type 1 pilus expression, if any, is gained by using an unlinked recombinase instead of or in addition to regulating FimB and FimE; and (vi) is the regulation of the *fimS* switch important for the evolution or maintenance of the unlinked recombinases, particularly since they are not conserved in all E. coli strains (and thought to be on at least partially mobile elements)? We have used whole-genome sequencing, combined with overexpression of individual recombinases, to answer the first two of these questions. We found that the *fim* recombinases are very specific and that, at least for CFT073 and UTI89, there are no other inversion targets for any of the recombinases aside from those already known. This therefore limits the complexity of questions iii and iv above while further shedding light on question vi regarding the importance of type 1 pili and their regulation in E. coli.

Positive verification of a new inversion locus is relatively straightforward once the locus is known, and two recent studies have used whole-genome sequencing (with Illumina and PacBio data) to achieve accurate quantification of *fimS* inversion percentages under different conditions (42, 43). However, to truly establish the specificity of the *fim* recombinases, a strong negative predictive value is required when analyzing whole-genome sequencing data (alternatively, a low noise level). With SVRE, we have improved the analysis of insert read lengths from paired-end short read sequencing data, leading to both sensitive and specific detection of inversions throughout the genome. The key analytical contribution of SVRE is to apply a theoretically optimal measure of differences in distributions (from an information theory perspective) that can then be related to the underlying structure of the genome. More explicitly, currently popular second-generation sequencing technology generates paired-end reads; the reads within each pair are separated by a certain distance, determined by the library preparation. Importantly, the distribution of distances should not depend on the DNA sequence itself (or location on the genome). Therefore, we can use a comparison of local versus global insert length distributions to identify when the genome structure does not match our expectation. This type of analysis is also referred to as anomaly detection, in which relative entropy is a commonly used technique (44). Many other SV detection programs use the same underlying idea, in which anomalous insert lengths are equated to variation in the genome structure, but they make the assumption that the read length distribution is normal (45, 46). Our use of relative entropy in SVRE therefore brings several key advantages: (i) generality to any distribution of insert lengths (which may change depending on how library preparation and size selection are done), (ii) elimination of parameters required to tune the program (such as specifying the expected mean and variance of the assumed normal distribution), (iii) utilization of information contained in “concordant” reads that are within the bulk of the expected distribution (these are still used in the calculation of relative entropy), and (iv) removal of the need for a cutoff for the number of “discordant” reads.

From a practical point of view, we find that SVRE produces generally low background signals for most of the genome, from which known SVs clearly stand out (between 3.5 and 4.5 Mbp) (Fig. 2). To make an assessment of the value of using information theory to analyze read length distributions, we reanalyzed our sequencing data with five other commonly used programs, including GASVPro (47), SVDetect (46), Pindel (48), breseq (49), and DELLY (45) (Fig. S3). In general, DELLY showed the greatest agreement with SVRE, while GASVPro had the least overlap. Some of these algorithms, such as GASVPro and Pindel, produced many more predictions than SVRE and required applying a cutoff to allele depth in order reduce the calls to a manageable number. A clear advantage of SVRE is that it enables a simple visualization of the relative entropy (Fig. 2 and 3), in addition to providing a list of SV predictions. The connection between DNA structure and relative entropy provides a natural priority ranking for validation and study of individual SVs. Use of SVRE on UTI89 and CFT073 thus allowed us to identify all previously known targets of the Fim recombinases as invertible sequences in the genome. We also identified several SVs that were unrelated to the Fim recombinases. Finally, the good signal-to-noise ratio provides confidence that under the conditions tested, we indeed found no additional invertible elements in the entire genome.

10.1128/mSphere.00693-18.4 FIG S3 Comparison of SVRE calls to that of other SV prediction programs. SV predictions for UTI89 (A) and CFT073 (B) are listed in the first columns of each table. Whether that SV was detected in a given sample by a program is indicated by a filled box following the color code indicated in the key. Download FIG S3, PDF file, 0.4 MB. Copyright © 2019 Russell et al. 2019 Russell et al. This content is distributed under the terms of the Creative Commons Attribution 4.0 International license.

SVRE detected various SVs that occurred independently of Fim recombinase activity. These included prophage duplications and deletions, types of SVs that were detected in both uropathogenic strains (Fig. S1 and S2). Apparent deletion of the prophage is likely due to excision from spontaneous prophage induction (50), while duplication could be due to recombination involving the flanking attachment sites or between two copies of the phage during theta replication (51). Other SVs were detected only in UTI89, such as an inversion associated with *asnW* and *asnV* (Fig. S1F), which have identical sequences. Interestingly, the orientation of the genes between *asnW* and *asnV* is inverted in CFT073 in comparison to that in UTI89 and other E. coli strains (52), which indicates that this may be a common, and possibly dynamic, inversion. Finally, two inversions occurred within prophage in UTI89 (Fig. S1D and E), one of which was adjacent to a phage invertase.

Among the previously identified inversion loci, we found that *ipuS* could be inverted by FimB, both in its native context in the CFT073 chromosome (Fig. 3) and when the *ipuS* switch was inserted into a plasmid (Fig. 4). In contrast, the original work identifying *ipuS* concluded that FimB was not capable of inverting *ipuS* (34). We did find that of the five Fim recombinases, FimB inverted *ipuS* in either direction with the lowest efficiency (Fig. 4B and C), making its effects more difficult to detect. Combined with differences in the chosen promoters to drive FimB expression, this possibly accounts for the discrepancy between the two studies. Our results also confirm that *ipuS* orientation regulates expression of *ipuR* and *upaE*, while clarifying that the *dsd* operon is not regulated by *ipuS* (Fig. 4D). Interestingly, FimE strongly drove inversion from OFF to ON in the MDS42 background (Fig. 4B) but not in the CFT073 background (Fig. 4C). Of note, while traditionally FimE was thought to mediate inversion only in the ON-to-OFF direction, FimE has been noted to mediate OFF-to-ON inversion under some conditions in different strains (42, 53). Therefore, these FimE results could be due to the allele of FimE or other strain-dependent differences.

We note that these experiments were performed using inducible-expression plasmids which allowed for recombinase overexpression, which is a commonly used strategy to increase inversion rates and facilitate identification (31, 33, 34, 42, 54, 55). However, future work employing native expression levels will be needed to determine physiological switching levels. Furthermore, these initial studies were performed in rich media at 37°C. As growth conditions are known to have a major impact on *fimS* inversion frequencies (38), future experiments employing different conditions will help delineate how these structural variations occur in various niches. Finally, we have focused in this study on genomic events; it remains possible that other layers of regulation may be influenced by these recombinases (such as transcription or posttranscriptional events).

It is remarkable that inversion of *fimS* is regulated by five Fim recombinases that invert only *fimS* or one or two other switches. The convergence at *fimS* suggests a potentially intricate coordination to control type 1 pilus expression; presumably this facilitates optimal host colonization or adhesion in some other evolutionarily relevant environment. The genetic context for these recombinases may provide some hints as to how *fimS* regulation by both “core” and “accessory” recombinases has evolved. FimB and FimE are considered to be core recombinases since they are encoded adjacent to *fimS* and are present in nearly all E. coli strains (56). In contrast, the accessory recombinases FimX, IpuA, and IpuB are encoded at distal locations on two different pathogenicity islands. FimX is encoded adjacent to *hyxS*, while IpuA and IpuB are encoded adjacent to *ipuS*. Therefore, it seems likely that the original role of FimX was to regulate *hyxS*, while IpuA and IpuB originally regulated *ipuS*. We speculate that subsequent to UPEC acquiring the pathogenicity islands containing these recombinases, the recombinases began to regulate *fimS* in addition to their cognate switch, and that this additional layer of regulation has given UPEC some sort of advantage. This idea is supported by the observation that *fimX* is enriched in UPEC strains (83.2%) compared to commensals (36%) (56). However, *ipuA* and *ipuB* are found at low levels in roughly equal proportions among UPEC (23.7%) and commensal (15%) strains alike (56). How these three switches, whose IRs differ in length and sequence, could be regulated by multiple recombinases is still not clear and an area for further investigation. FimB and FimE have been shown to bind to *fimS* at the IRs at half-sites that overlap and flank the IRs (57). Therefore, one would hypothesize that the IRs and their surrounding sequences would be quite similar. There is some alignment observed between *ipuS* and *fimS* and between *ipuS* and *hyxS* (34). However, the alignment between *fimS* and *hyxS* is poor, despite the fact that FimX is able to facilitate recombination at both switches (31–33). It thus remains an open question how the Fim recombinases recognize these IRs with apparently dissimilar sequences.

The fact that additional recombinases regulate *fimS* supports the notion that proper type 1 pilus expression is important to the evolutionary success of UPEC. Indeed, expression of type 1 pili is regulated by several factors which modulate either inversion of *fimS* or transcriptional activity, including LrhA, PapB, SfaB, H-NS, IHF, RpoS, ppGpp, DksA, *leuX*, Lrp, and CRP (reviewed in reference 15). Many of these factors coordinate type 1 pilus expression with other virulence factors (e.g., P pili and flagella) or with the metabolic state of the cell. Environmental cues are also critical for modulation of expression. For example, both expression and function of type 1 pili are decreased in urine but increased in the vicinity of host cells to which they can adhere (58), suggesting that type 1 pilus expression may be programmed for induction in specific niches.

The evolutionary importance of type 1 pili is highlighted by the observation of positive selection on the FimH adhesin, which results in tuning the conformational flexibility of the protein, leading to modulation of the dynamics of binding to the surface of bladder epithelial cells (59–63). Selection may also occur at the transcriptional level, as a spontaneous mutation in LrhA increases expression of type 1 pili and correlates with increased virulence in sepsis (64). Of note, proper regulation may in some cases include downregulation of type 1 pilus expression at appropriate times, which is also supported by the regulatory mutations seen in enterohemorrhagic E. coli (EHEC) (to lock the *fimS* switch in the OFF orientation) (65), the widespread inactivation of *fimB* in the ST131 E. coli lineage via an insertion sequence (42), and the strong positive selection on *fimA* (thought to be due to immune evasion) (66). Downregulation may also explain the finding of low type 1 pilus expression in bacteria in the urine of some human UTI patients (67–69), though variation in the interaction between different hosts and pathogens during infection is another possibility (70). Here we have provided additional data that argue that type 1 pili are important to the success of E. coli, particularly UPEC, suggesting that current efforts to target type 1 pilus function to prevent and treat UTI represent a rational antivirulence strategy.

## MATERIALS AND METHODS

### Bacterial strains.

All strains utilized in this study are listed in Table S1. Creation of knockout strains was done using lambda red recombination (71) with 50-bp flanking sequences as described before (72). Primers used for recombination are listed in Table S2.

10.1128/mSphere.00693-18.5 TABLE S1 Strains utilized in this work. If the strain was part of a previous study, the appropriate reference is given. Download Table S1, PDF file, 0.09 MB. Copyright © 2019 Russell et al. 2019 Russell et al. This content is distributed under the terms of the Creative Commons Attribution 4.0 International license.

10.1128/mSphere.00693-18.6 TABLE S2 Primers used for strain creation, SV validation, and qRT-PCR. The table lists primer sets used to detect SVs, create knockout mutant strains, and measure gene expression. Download Table S2, PDF file, 0.06 MB. Copyright © 2019 Russell et al. 2019 Russell et al. This content is distributed under the terms of the Creative Commons Attribution 4.0 International license.

### Preparation of sequencing data.

Overnight cultures were diluted 1:100 into LB broth containing chloramphenicol (20 μg/ml) and were incubated with shaking at 25°C for 24 h, then diluted 1:1,000 into fresh medium supplemented with chloramphenicol and arabinose (0.5%), and incubated for another 24 h. After the 48-h growth period, genomic DNA (gDNA) was extracted and prepared for Illumina sequencing. For UTI89, the library was prepared using standard techniques, including shearing, end repair, size selection, PCR, and purification with AMPure XP beads; sequencing was performed on an Illumina HiSeq 2000 machine as paired reads with a length of 76 bp. The CFT073 libraries were made using the Illumina TruSeq DNA library prep kit v2 and were sequenced on the Illumina MiSeq as paired reads of a length of 150 bp.

### Development of SVRE.

We developed SVRE to improve on existing strategies used in SV detection, particularly those which make use of insert length distributions. When mapped to a perfect reference (i.e., not containing an SV), paired reads will map on opposite strands and at a distance determined by the insert size of the sequencing library, which is usually intentionally controlled during library preparation. Paired reads that map in this way are referred to as “concordant” pairs, while those that do not are “discordant.” One immediate strategy is to focus on discordant reads; clusters of discordant reads mapping to a particular region of the genome are then identified as a potential SV. However, distinguishing between these two classes is not always trivial, and appropriate cutoffs for how many discordant reads should be required to support a true SV are difficult to determine *a priori*. Programs such as GASVPro (47), SVDetect (46), DELLY (45), VariationHunter (73), and BreakDancer (74) and the read distribution module of LUMPY (75) define concordant reads as those whose mapping distances fall within a chosen range based on the expected mapping distance and the standard deviation. In other words, library preparation is assumed to generate a roughly normal distribution of read insert lengths. Another drawback to this approach is that concordant reads are discarded and any information that concordant reads could supply for predicting SVs (such as differences in their length distribution) is lost.

Another strategy that avoids this concordant/discordant differentiation considers the overall distribution of mapping distances. By looking at histograms of mapping distances, changes from the expected distribution can be detected by a number of methods, including statistical tests (χ^2^, K-S test, *t* test, Z-test, etc.) or by using classification algorithms (such as support vector machines). Existing algorithms that utilize this distribution comparison strategy include SVM^2^ (76) and MoDIL (77).

SVRE also uses a distribution comparison strategy. We choose the global insert length distribution as an empirical null model; implicitly, we are assuming that SVs are rare overall and therefore have a minimal global effect on the insert length distribution. We then compare the distribution of a local window to this global distribution using relative entropy (Kullback-Leibler divergence, relative information content, or information divergence/gain). In information theory, relative entropy is a measure of the divergence between two “information” distributions (35). This is strongly related to concepts about signal encoding and compression, in which entropy is known to define an optimal theoretical lower limit for compressed or encoded message size. With respect to SV detection, to the extent that information is carried within insert length distributions, we suggest that relative entropy is a potentially optimal statistic for quantifying how different a local distribution is from the global null distribution, though we have not formally proven this.

Details about the implementation of SVRE can be found in Text S1. SVRE was written in Perl and is available for download at https://github.com/swainechen/svre.

### Structural variation prediction with other software.

GASVPro version 1.2 (47), SVDetect version 0.8b (46), Pindel version 0.2.5b9 (48), breseq version 0.33.1 (49), and DELLY version 0.7.8 (45) were run according to the instructions provided by the developers. Fastq files were used as the input for breseq, whereas the other programs required sorted, paired-end bam files which were produced using BWA-MEM (78) and SAMtools (79). Any additional pre- and postprocessing steps, as well as analysis of the output, were performed *ad hoc* with Python.

### PCR to confirm structural variations.

The primers utilized to validate predicted SVs are listed in Table S2 and were designed according to the specific SV type as outlined in Fig. S1A to C. Validation was performed with cells grown for 48 h at 25°C with passaging at 24 h and cells grown for 7 h at 37°C. The cells were grown in LB broth with arabinose to induce expression of recombinases. PCR was performed with cells from a freshly grown culture or with gDNA isolated from the culture using a DNeasy blood and tissue kit (Qiagen). DreamTaq polymerase (Thermo Scientific) was used for the PCRs according to the manufacturer’s instructions, with deoxynucleoside triphosphates (dNTPs) at a concentration of 2 mM and primers at 0.5 μM, and the following thermocycler settings: 95°C for 3 min; 30 cycles of 95°C for 30 s, 55°C for 30 s, and 72°C for 1 min; and 72°C for 5 min.

### Cloning.

The vectors pSLC-372 and pSLC-373 contain the *ipuS* switch in the OFF and ON positions, respectively, cloned into the BamHI and SacI sites of pUC19. To obtain *ipuS* DNA in both orientations, *ipuS* was amplified from CFT073/pBAD-ipuA cells induced with arabinose. Plasmids encoding for Fim recombinases were made by amplifying the recombinase from the genomic DNA of either UTI89 or CFT073 and cloning it into the SacI and XbaI sites of pBAD33. The same FimB plasmid was used for both strains given that the *fimB* sequence is identical in the two genomes. These plasmids, along with the primers used for making them, are listed in Table S3. Phusion polymerase (New England BioLabs) was used to amplify insert DNA according to the manufacturer’s instructions, with dNTPs at a concentration of 2 mM and primers at 0.5 μM and the following thermocycler settings: 98°C for 30 s; 30 cycles of 98°C for 10 s, ∼60°C for 20 s, and 72°C for 20 s/kb of amplicon length; and 72°C for 5 min. Plasmids were isolated from cells using the QIAprep Spin miniprep kit (Qiagen).

10.1128/mSphere.00693-18.7 TABLE S3 Plasmids utilized in this work. For each plasmid, either a reference is given or the primers that were used in the creation of the plasmid are listed. Download Table S3, PDF file, 0.01 MB. Copyright © 2019 Russell et al. 2019 Russell et al. This content is distributed under the terms of the Creative Commons Attribution 4.0 International license.

### Quantification of *ipuS* orientation.

Overnight cultures were diluted 1:100 into 2 ml of LB broth supplemented with chloramphenicol (20 µg/ml) and arabinose (0.5%) and grown with shaking for 7 h at 37°C. A PCR was then performed to amplify across the *ipuS* switch using primers cwr175 and cwr178 to amplify from the genome or primers M13F and M13R to amplify from the plasmids pSLC-372 and pSLC-373 (Table S2). PCR was performed with DreamTaq as described above. The resulting product was digested with PacI, which has only one site in the PCR product that is located within *ipuS*. This digestion reaction results in two bands that differ in size depending on the orientation of the switch. The digest reactions were run on a 2% gel and imaged, and the densities of one OFF orientation band and one ON orientation band were quantified using ImageJ FIJI. The total density of the two bands was set to 100% and the percent ON versus OFF was then calculated.

### RT-qPCR.

Overnight cultures of CFT073 carrying pBAD33, pBAD-ipuA, or pBAD-ipuB were subcultured 1:100 into 10 ml of LB broth with chloramphenicol (20 µg/ml) in a 100-ml flask and were grown with shaking for 3 h at 37°C. Arabinose was then added to a final concentration of 0.5%, and the cells were allowed to incubate for another hour, at which point 0.5 ml of culture was added to 1 ml of RNAprotect bacterial reagent and the cells were lysed using proteinase K and lysozyme. RNA was isolated using the RNeasy minikit, and DNA was removed with DNase I digestion. The SuperScript II RT kit was used to make cDNA. For each sample, a control reaction was run that lacked reverse transcriptase to check for DNA contamination during the qPCR reactions.

Primers employed in the qPCR are listed in Table S2. A control lacking cDNA was included for each pair of primers, in addition to the reactions with and without reverse transcriptase for each sample. KAPA SYBR FAST qPCR master mix was used along with 0.5 µM each primer and ROX Low. The reactions were run on the ViiA 7 real-time PCR system with the following program: 95°C for 3 min, followed by 40 cycles of 95°C for 3 s and 60°C for 20 s. The data were analyzed using the threshold cycle (ΔΔ*C_T_*) method with 16S acting as a reference gene and the pBAD33 sample as the reference sample (80). Differences between sample Δ*C_T_* values were tested using an unpaired, two-tailed *t* test.
