# Supplementary material for: Comprehensive Identification of Fim-Mediated Inversions in Uropathogenic Escherichia coli with Structural Variation Detection Using Relative Entropy
Source: mSphere. 2019 Apr 10;4(2):e00693-18. doi: 10.1128/mSphere.00693-18 (PMC6458436; doi:10.1128/mSphere.00693-18)
Supplement: TEXT S1 [file mSphere.00693-18-s0001.pdf]

Implementation of SVRE

SVRE takes paired reads mapped to a reference, windows them, and calculates the RIC score (i.e. relative entropy) and a resampling-based p-value for each window, as detailed below.

SVRE ensures that unambiguous mapping data is used for SV detection. First, SVRE accepts two input BAM files, one for each sequence of the read pair (typically R1 and R2 for Illumina sequencing); in these BAM files, all reads must have been mapped singly without any pair information. This avoids the possibility of mismapping that can occur with a pair-rescue mapping strategy. Second, SVRE filters input to accept only those paired reads that map uniquely with no mismatch to the reference genome. Reads mapping to the positive strand of the genome are assigned a positive coordinate position, while those mapping to the negative strand are assigned a negative position.

Each read pair (and thus each read) has an associated mapping distance. The expected value of this mapping distance is the mean insert size of the fragments generated during library preparation. To track relative orientation, we establish a coordinate system in which, by convention, concordant read pairs mapping in the expected orientation (opposite strands for Illumina, same strand for SoLID) have a positive mapping distance, and those in the opposite orientation have a negative mapping distance. The entire set of mapping distances is tabulated into a histogram whose bin sizes are set at half the expected (mean) mapping distance–this is referred to as the global histogram.

The reads are then binned based on their mapping coordinate into non-overlapping windows of equal coverage (referred to as n). Each window then contains an equal number of mapping distances. The mapping distances in each window are then tabulated into a histogram with the same bin sizes as the global histogram – this is referred as the local histogram. Note that

some read pairs may have both reads mapping within the same coverage window. In this case, their distances are counted twice (once for each read of the pair) for the local histogram. This ensures equal weight, as the mapping distance for other read pairs is also counted twice, one in each of two coverage windows.

Finally, the relative entropy of the local histograms relative to the global histogram is calculated using the following formula where R is the relative entropy for a window, and L and G are the local and global distributions, respectively.

$$R = \sum_{i}^{i=1\ to\ n} L(i)\ log_2\left(\frac{L(i)}{G(i)}\right)$$

P-values are calculated by resampling; 10^6 subsets of n mapping distances are sampled with replacement from the entire set of read pair mapping distances. The relative entropy of the histograms of these resampled windows to the global histogram is then used to assign p-values. Multiple testing corrections are done using the false discovery rate ($\alpha = 0.05$) method across all the windows in all chromosomes in the dataset. Finally, a q-value calculated based on the corrected p-values is used as the cutoff, with the maximum allowed q-value set to 0.05. Thus, the cutoff is appropriate for each dataset depending on the p-value distribution for that dataset.

SVRE creates several output files that aid with downstream analysis. One file (<library>_re.txt) contains the relative entropy score and p-value for each window and is the basis for the <library>_graph.png figure produced by SVRE showing the relative entropy peaks across the genome. A second file (<library>_SV.txt) lists the windows with significant p-values ("Individual SV calls") as well as SV predictions made by SVRE ("Combined SV calls"). More detail for each window with a significant relative entropy is given by <library>_detail.txt.
