# Supplementary material for: Comprehensive Identification of Fim-Mediated Inversions in Uropathogenic Escherichia coli with Structural Variation Detection Using Relative Entropy
Source: mSphere. 2019 Apr 10;4(2):e00693-18. doi: 10.1128/mSphere.00693-18 (PMC6458436; doi:10.1128/mSphere.00693-18)
Supplement: FIG S1 [file mSphere.00693-18-sf001.pdf]

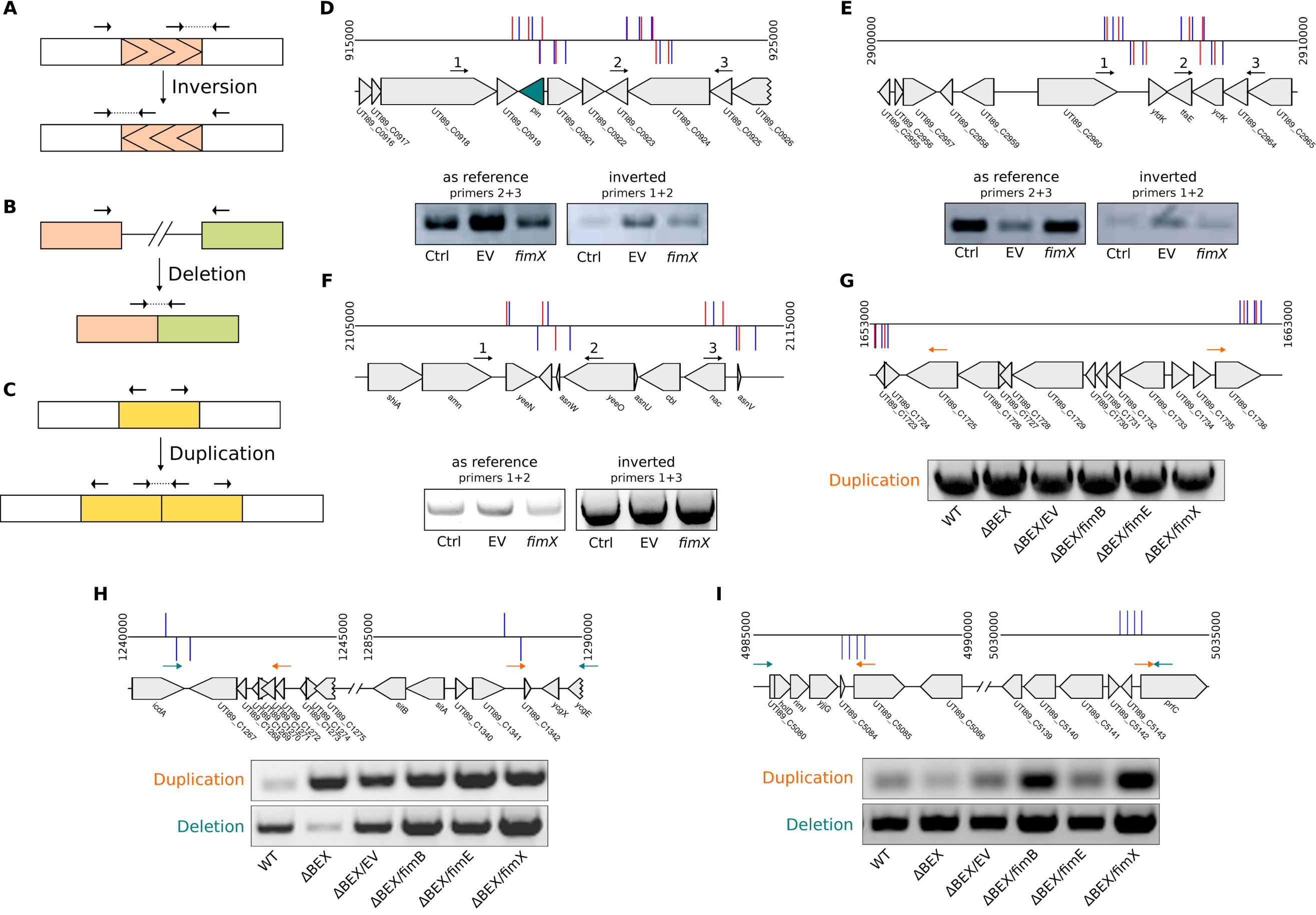

Figure S1. Confirmation of novel structural variations in UTI89. A PCR strategy was employed that was specific to each SV type. (A) For inversions, two sets of primers were used. One set produces a band when the invertible element is in the orientation found on the reference genome. In contrast, the other set produces a band if there is an inversion event. (B) Deletions were detected by using distant primer sets that only produce a band if the intervening sequence is deleted, bringing the priming sites closer together. (C) Duplications were detected using outward facing primer pairs that produce a band only if a tandem duplication event occurs. (D-I) For each SV, the leftmost coordinate of significant windows called by SVRE are represented by red (UTI89/pBAD-fimB) and blue (UTI89/pBAD-fimE) lines. The primers used to confirm the predicted SVs are depicted on the schematic of the neighboring genes, and the gels that resulted from the use of those primers are shown below. (D-F) Confirmation of inversions at (D) 0.9 Mb, (E) 2.1 Mb, and (F) 2.9 Mb were performed in UTI89 ("Ctrl"), UTI89/pBAD33 ("EV"), and UTI89/pBAD-fimX ("*fimX*") cells. The linked phage invertase *pin* is highlighted in (A). (G-I) Confirmation of (G) a prophage deletion at 1.6 Mb, prophage duplication and deletions at (H) 1.2 Mb and (I) 5.0 Mb. The PCR was performed using WT UTI89 as well as UTI89Δ*fimB*Δ*fimE*Δ*fimX* ("ΔBEX").
