# Supplementary material for: Comprehensive Identification of Fim-Mediated Inversions in Uropathogenic Escherichia coli with Structural Variation Detection Using Relative Entropy
Source: mSphere. 2019 Apr 10;4(2):e00693-18. doi: 10.1128/mSphere.00693-18 (PMC6458436; doi:10.1128/mSphere.00693-18)
Supplement: FIG S2 [file mSphere.00693-18-sf002.pdf]

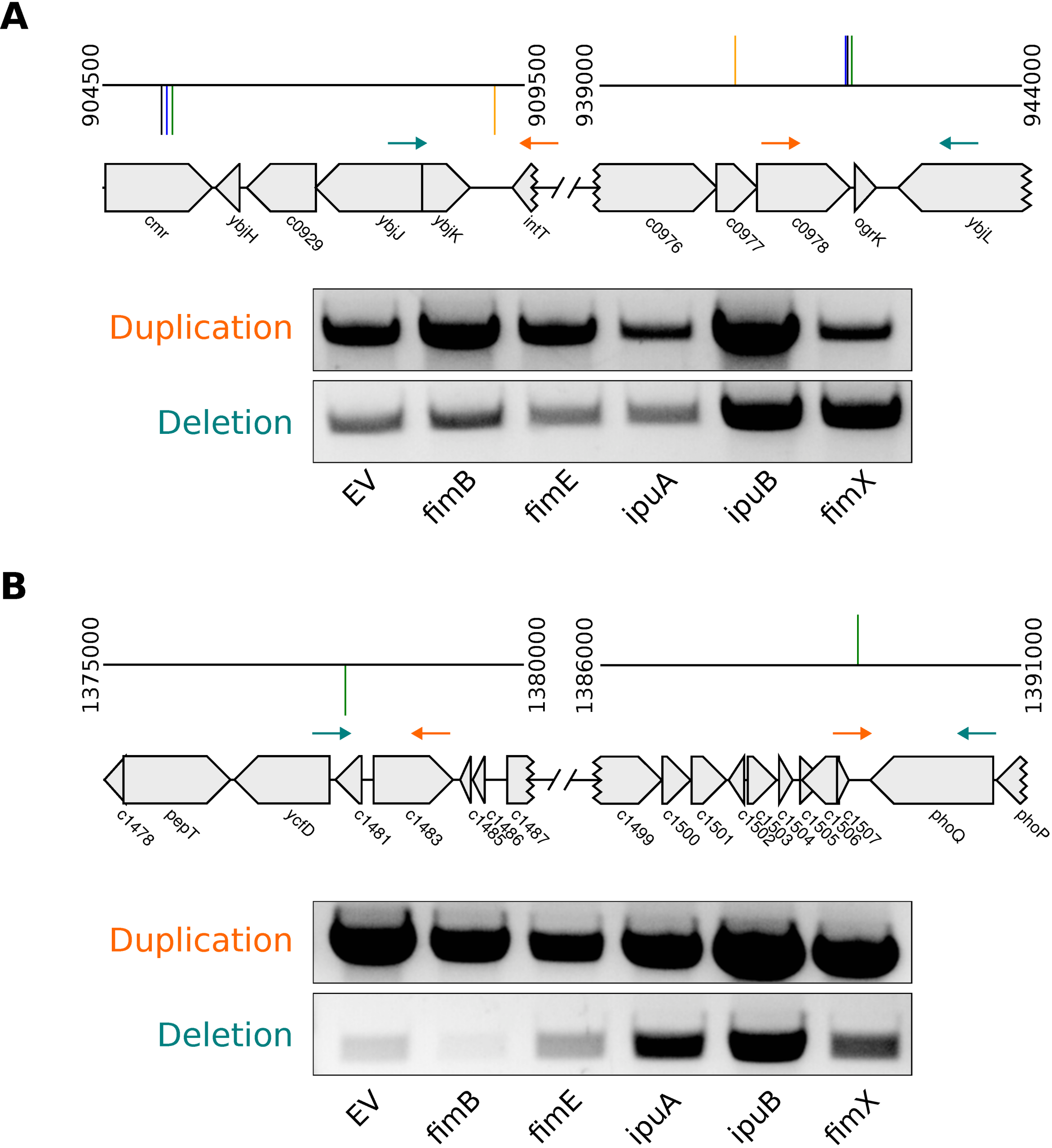

Figure S2. Confirmation of novel structural variations in CFT073. For each SV, the leftmost coordinate of significant windows called by SVRE are represented by red (pBAD-fimB), black (pBAD-fimE), orange (pBAD-ipuA), green (pBAD-ipuB), and blue (pBAD-fimX) lines. The primers used to confirm the predicted SVs are depicted on the schematic of the neighboring genes, and the gels that resulted from the use of those primers are shown below. Confirmation of the SVs was performed in CFT073 carrying either pBAD33 ("EV") or plasmids encoding the various recombinases. (A) Detection of duplication and deletion of phage at 0.9 Mb and (B) a phage at 1.3 Mb.
