# Supplementary material for: Comprehensive Identification of Fim-Mediated Inversions in Uropathogenic Escherichia coli with Structural Variation Detection Using Relative Entropy
Source: mSphere. 2019 Apr 10;4(2):e00693-18. doi: 10.1128/mSphere.00693-18 (PMC6458436; doi:10.1128/mSphere.00693-18)
Supplement: FIG S3 [file mSphere.00693-18-sf003.pdf]

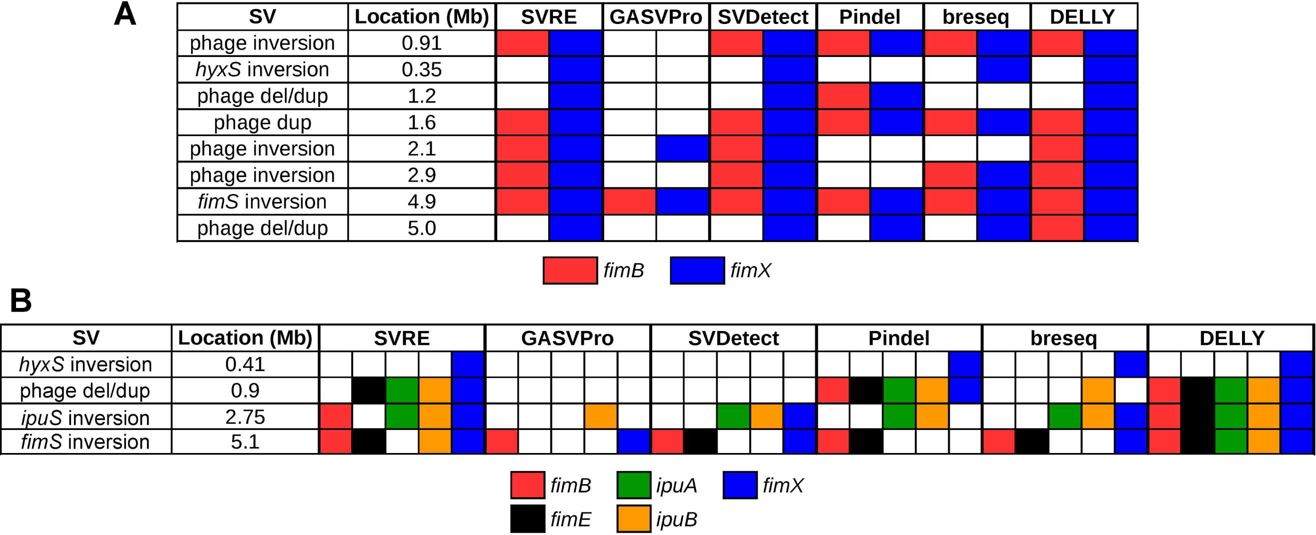

Figure S3. Comparison of SVRE calls to that of other SV prediction programs. SV predictions for (A) UTI89 and (B) CFT073 are listed in the first columns of each table. Whether that SV was detected in a given sample by a program is indicated by a filled box following the color code indicated in the legend.
