# Supplementary material for: Comprehensive Identification of Fim-Mediated Inversions in Uropathogenic Escherichia coli with Structural Variation Detection Using Relative Entropy
Source: mSphere. 2019 Apr 10;4(2):e00693-18. doi: 10.1128/mSphere.00693-18 (PMC6458436; doi:10.1128/mSphere.00693-18)
Supplement: TABLE S1 [file mSphere.00693-18-st001.pdf]

**Strain Name**	**Strain Number**	**Reference**
UTI89		1
UTI89/pBAD33	SLC-625	This work
UTI89/pSLC-145 (*fimX*)	SLC-627	This work
UTI89/pSLC-146 (*fimB*)	SLC-628	This work
UTI89Δ*fimB*Δ*fimE*Δ*fimX* *fimS*$_{OFF}$	SLC-533	This work
UTI89Δ*fimB*Δ*fimE*Δ*fimX* *fimS*$_{OFF}$/pBAD33	SLC-676	This work
UTI89Δ*fimB*Δ*fimE*Δ*fimX* *fimS*$_{OFF}$/pSLC-145 (*fimX*)	SLC-679	This work
UTI89Δ*fimB*Δ*fimE*Δ*fimX* *fimS*$_{OFF}$/pSLC-146 (*fimB*)	SLC-677	This work
UTI89Δ*fimB*Δ*fimE*Δ*fimX* *fimS*$_{OFF}$/pSLC-147 (*fimE*)	SLC-678	This work
CFT073		2
CFT073/pBAD33	SLC-665	This work
CFT073/pSLC-289 (*fimE*)	SLC-667	This work
CFT073/pSLC-290 (*ipuA*)	SLC-668	This work
CFT073/pSLC-291 (*ipuB*)	SLC-669	This work
CFT073/pSLC-292 (*fimX*)	SLC-670	This work
CFT073/pSLC-146 (*fimB*)	SLC-666	This work
CFT073Δ*ipuA::kan*/pBAD33	SLC-1102	This work
CFT073Δ*ipuA::kan*/pSLC-289	SLC-1104	This work
CFT073Δ*ipuA::kan*/pSLC-290	SLC-1105	This work
CFT073Δ*ipuA::kan*/pSLC-291	SLC-1106	This work
CFT073Δ*ipuA::kan*/pSLC-292	SLC-1107	This work
CFT073Δ*ipuA::kan*/pSLC-146	SLC-1103	This work
CFT073Δ*ipuB::kan*/pBAD33	SLC-1108	This work
CFT073Δ*ipuB::kan*/pSLC-289	SLC-1110	This work
CFT073Δ*ipuB::kan*/pSLC-290	SLC-1111	This work
CFT073Δ*ipuB::kan*/pSLC-291	SLC-1112	This work
CFT073Δ*ipuB::kan*/pSLC-292	SLC-1113	This work
CFT073Δ*ipuB::kan*/pSLC-146	SLC-1109	This work
CFT073Δ*ipuAB::kan*/pBAD33	SLC-1114	This work
CFT073Δ*ipuAB::kan*/pSLC-289	SLC-1116	This work
CFT073Δ*ipuAB::kan*/pSLC-290	SLC-1117	This work

CFT073Δ*ipuAB::kan*/pSLC-291	SLC-1118	This work
CFT073Δ*ipuAB::kan*/pSLC-292	SLC-1119	This work
CFT073Δ*ipuAB::kan*/pSLC-146	SLC-1115	This work
MDS42		3
MDS42/pSLC-372/pBAD33	SLC-1122	This work
MDS42/pSLC-372/pSLC-289	SLC-1124	This work
MDS42/pSLC-372/pSLC-290	SLC-1125	This work
MDS42/pSLC-372/pSLC-291	SLC-1126	This work
MDS42/pSLC-372/pSLC-292	SLC-1127	This work
MDS42/pSLC-372/pSLC-146	SLC-1123	This work
MDS42/pSLC-373/pBAD33	SLC-1128	This work
MDS42/pSLC-373/pSLC-289	SLC-1130	This work
MDS42/pSLC-373/pSLC-290	SLC-1131	This work
MDS42/pSLC-373/pSLC-291	SLC-1132	This work
MDS42/pSLC-373/pSLC-292	SLC-1133	This work
MDS42/pSLC-373S/pSLC-146	SLC-1129	This work

References:

1. S. L. Chen, C. S. Hung, J. Xu, C. S. Reigstad, V. Magrini, A. Sabo, D. Blasiar, T. Bieri, R. R. Meyer, P. Ozersky, J. R. Armstrong, R. S. Fulton, J. P. Latreille, J. Spieth, T. M. Hooton, E. R. Mardis, S. J. Hultgren, and J. I. Gordon, Proc Natl Acad Sci, 103(15):5977-82, 2006, doi:10.1073/pnas.0600938103
2. R. A. Welch, V. Burland, G. Plunkett, P. Redford, P. Roesch, D. Rasko, E. L. Buckles, S. R. Liou, A. Boutin, J. Hackett, D. Stroud, G. F. Mayhew, D. J. Rose, S. Zhou, D. C. Schwartz, N. T. Perna, H. L. Mobley, M. S. Donnenberg, and F. R. Blattner, Proc Natl Acad Sci, 99(26):17020-4, 2002, doi:10.1073/pnas.252529799
3. G. Pósfai, G. Plunkett, T. Fehér, D. Frisch, G. M. Keil, K. Umenhoffer, V. Kolisnychenko, B. Stahl, S. S. Sharma, M. de Arruda, V. Burland, S. W. Harcum, F. R. Blattner, Science, 312(5776):1044-6, 2006, doi:10.1126/science.1126439
