# Supplementary material for: Comprehensive Identification of Fim-Mediated Inversions in Uropathogenic Escherichia coli with Structural Variation Detection Using Relative Entropy
Source: mSphere. 2019 Apr 10;4(2):e00693-18. doi: 10.1128/mSphere.00693-18 (PMC6458436; doi:10.1128/mSphere.00693-18)
Supplement: TABLE S2 [file mSphere.00693-18-st002.pdf]

Application	Primers
Validation of inversion at 0.9 Mb in UTI89	CCCTCCTGACCGGATCAATG<br>GCCCAGCCGTAGCTGTGAGT<br>GCGAAGGTGGGCGTAATGAG
Validation of duplication at 1.2 Mb in UTI89	ATATCCGCACCCAGCAGCAT<br>TCGCGGAGAGAAAACGGAAG
Validation of deletion at 1.2 Mb in UTI89	GCCCGGTAAAGAGGCCATTC<br>TACCAACTGGCGCGTGAAGA
Validation of duplication at 1.6 Mb in UTI89	TGGAACTGGCTGGGGAATGT<br>TTCGAACCGGCTCCATTGAT
Validation of inversion at 2.1 Mb in UTI89	GCAACGTGCGCTTTATGACG<br>CTGGATACCCCAGCCACAGG<br>AAGGTTGGGGGTAGGGCAGA
Validation of inversion at 2.9 Mb in UTI89	CGCTTGCATCAATGCTCCAC<br>CTGTGCTTCTGCCGCGTCTA<br>CGTCTGTTGTCGGCACTGCT
Validation of duplication at 5.0 Mb in UTI89	ACGTTCCTTTCCAACGACTG<br>GCGAAAAGAAGAACCTTTGC
Validation of deletion at 5.0 Mb in UTI89	ATCACTGGAAGGCGCTCAGG<br>GCAGCCGATTTTCAGCTCGT
Validation of duplication at 0.9 Mb in CFT073	AACAAAACTCCGGCATTCAG<br>ACCCATGAGCAGATTGTTGA
Validation of deletion at 0.9 Mb in CFT073	ACCAAAACTAGCCCCAAAGA<br>TGCGCCTTCTTTGTTATTGG
Validation of duplication at 1.38 Mb in CFT073	CCTCAAATTGAAGAGGTCGG<br>AATCAGGAGGCGGATTAGTG
Validation of deletion at 1.38 Mb in CFT073	TTCTCCGAGATGATCGTAGC<br>GAAGTGCGGGAAGATGATGA
RT-qPCR: 16S	ATGACCAGCCACACTGGAACT<br>AGTATCAGATGCAGTTCCCAG
RT-qPCR: *upaE*	TTCTGCGCGGTTCTCAATCC<br>GGAAGCCGTATTGCGGTCAA
RT-qPCR: *ipuR*	TCTACGACGATGCGTCCACA<br>GGCTAAAAAGCCAGCCTGTCA
RT-qPCR: *ipuA*	TTTGCGTCACCTATCCCCCA<br>CCCGTGGTTATTTGTTTCCCGA
RT-qPCR: *ipuB*	AGGGTAAGCGAGCTTATTGCAC<br>GCATACCCACAACCATGCCT
RT-qPCR: *dsdC*	TAGAGGGCCGGGAATACAGC

	AGGCATCAGTCCTTCGCTCT
RT-qPCR: *dsdX*	GCATGGGGCCACTGGATATG<br>CAGCGGAATGGCCAGCTTTA
Detection of *ipuS* inversion from CFT073 genome	CACCTCCAGCCATTGATAAA<br>TCGTAATATCAGGCATACGG
Detection of *ipuS* inversion from pSLC-372 and pSLC-373 plasmids	TGTAAAACGACGGCCAGT<br>CAGGAAACAGCTATGAC
Creation of CFT073Δ*ipuA::kan*	ATTCTTTCATCATAGTTGACATGGCTATTTCATATAAATAGAGGTGATTTTGTGTAGGCTGGAGCTGCTTC<br>ATTGAATTAGAAGCAGTATAAATTACCGTATGCCTGATATTACGATGCCCTATGAATATCCTCCTTAG
Validation of CFT073Δ*ipuA::kan*	CCGTATGCCTGATATTACGA<br>TCATCATAGTTGACATGGCT
Creation of CFT073Δ*ipuB::kan*	CTGGTATTTATAGTTCATTTTTTAACTTAATTAATATAACCGATAATATTTGTGTAGGCTGGAGCTGCTTC<br>AAAAATGAGGGGCTAAAAGCCTCAATGAATTGAGGCCTTTAGAACAGGGGTATGAATATCCTCCTTAG
Validation of CFT073Δ*ipuB::kan*	CAATGAATTGAGGCCTTTAG<br>CAAGCAATAATACCAGGCTC
Creation of CFT073Δ*ipuAB::kan*	AAAAATGAGGGGCTAAAAGCCTCAATGAATTGAGGCCTTTAGAACAGGGGTGTGTAGGCTGGAGCTGCTTC<br>ATTGAATTAGAAGCAGTATAAATTACCGTATGCCTGATATTACGATGCCCTATGAATATCCTCCTTAG
Validation of CFT073Δ*ipuAB::kan*	CAATGAATTGAGGCCTTTAG<br>CCGTATGCCTGATATTACGA
