# Supplementary material for: Comprehensive Identification of Fim-Mediated Inversions in Uropathogenic Escherichia coli with Structural Variation Detection Using Relative Entropy
Source: mSphere. 2019 Apr 10;4(2):e00693-18. doi: 10.1128/mSphere.00693-18 (PMC6458436; doi:10.1128/mSphere.00693-18)
Supplement: TABLE S3 [file mSphere.00693-18-st003.pdf]

Name	Description	Primers or Reference
pBAD33	Empty vector plasmid	L. M. Guzman, D. Belin, M. J. Carson, J. Beckwith, J Bacteriol, 177(14):4121-30, 1995.
pSLC-145	UTI89 *fimX* gene cloned into SacI and XbaI sites of pBAD33	GATCCTAGGAGCTCAAGAAGGAGATATACATGTGTGCCTCTCTTTTTTTTACAACT<br>GATCCTAGTCTAGATTAACGAGGCTTTTTTTTCCA
pSLC-146	UTI89/CFT073 *fimB* gene cloned into SacI and XbaI sites of pBAD33	GATCCTAGGAGCTCAAGAAGGAGATATACATATGAAGAATAAGGCTGATAAC<br>GATCCTAGTCTAGACTATAAAACAGCGTGACGCTG
pSLC-147	UTI89 *fimE* gene cloned into SacI and XbaI sites of pBAD33	GATCCTAGGAGCTCAAGAAGGAGATATACATGTGAGTAAACGTCGTTATCTT<br>GATCCTAGTCTAGATCAAGCTTCTTCTCTTTTTAA
pSLC-289	CFT073 *fimE* gene cloned into SacI and XbaI sites of pBAD33	GATCCTAGGAGCTCAAGAAGGAGATATACATGTGAGTAAACGTCGTTATCT<br>GATCCTAGTCTAGATCAAACTTCTTCTCTTTTTAA
pSLC-290	CFT073 *ipuA* gene cloned into SacI and XbaI sites of pBAD33	GATCCTAGGAGCTCAAGAAGGAGATATACATATGCAAAACAGAAAATTTTT<br>GATCCTAGTCTAGACTATACGAGAATTTCTAAAC
pSLC-291	CFT073 *ipuB* gene cloned into SacI and XbaI sites of pBAD33	GATCCTAGGAGCTCAAGAAGGAGATATACATATGCGCAAATTTATTACTCA<br>GATCCTAGTCTAGATTATATCTTCTTTATATCTA
pSLC-292	CFT073 *fimX* gene cloned into SacI and XbaI sites of pBAD33	GATCCTAGGAGCTCAAGAAGGAGATATACATGTGTGCCTCTCTTTTTTTTACAACT<br>GATCCTAGTCTAGATTAACGAGGCTTTTTTTTCCA
pSLC-372	CFT073 *ipuS* in OFF orientation cloned into BamHI and SacI sites of pUC19	GCGGCGGATCCGACGCAAAAAAGCAACATTT<br>GCGGCGAGCTCTGAGGCACAGGTTTACAAAA
pSLC-373	CFT073 *ipuS* in ON orientation cloned into BamHI and SacI sites of pUC19	GCGGCGGATCCGACGCAAAAAAGCAACATTT<br>GCGGCGAGCTCTGAGGCACAGGTTTACAAAA
